# Supplementary material for: p85β regulates autophagic degradation of AXL to activate oncogenic signaling
Source: Nat Commun. 2020 May 8;11:2291. doi: 10.1038/s41467-020-16061-7 (PMC7210311; doi:10.1038/s41467-020-16061-7)
Supplement: Supplementary file 1 — Supplementary Information [file 41467_2020_16061_MOESM1_ESM.pdf]

Supplementary information

p85β regulates autophagic degradation of AXL to activate oncogenic signaling

Ling Rao, Victor CY Mak, Yuan Zhou, Dong Zhang, Xinran Li, Chloe CY Fung, Rakesh Sharma, Chao Gu, Yiling Lu, George L Tipoe, Annie NY Cheung, Gordon B Mills, Lydia WT Cheung

Supplementary Figure 1

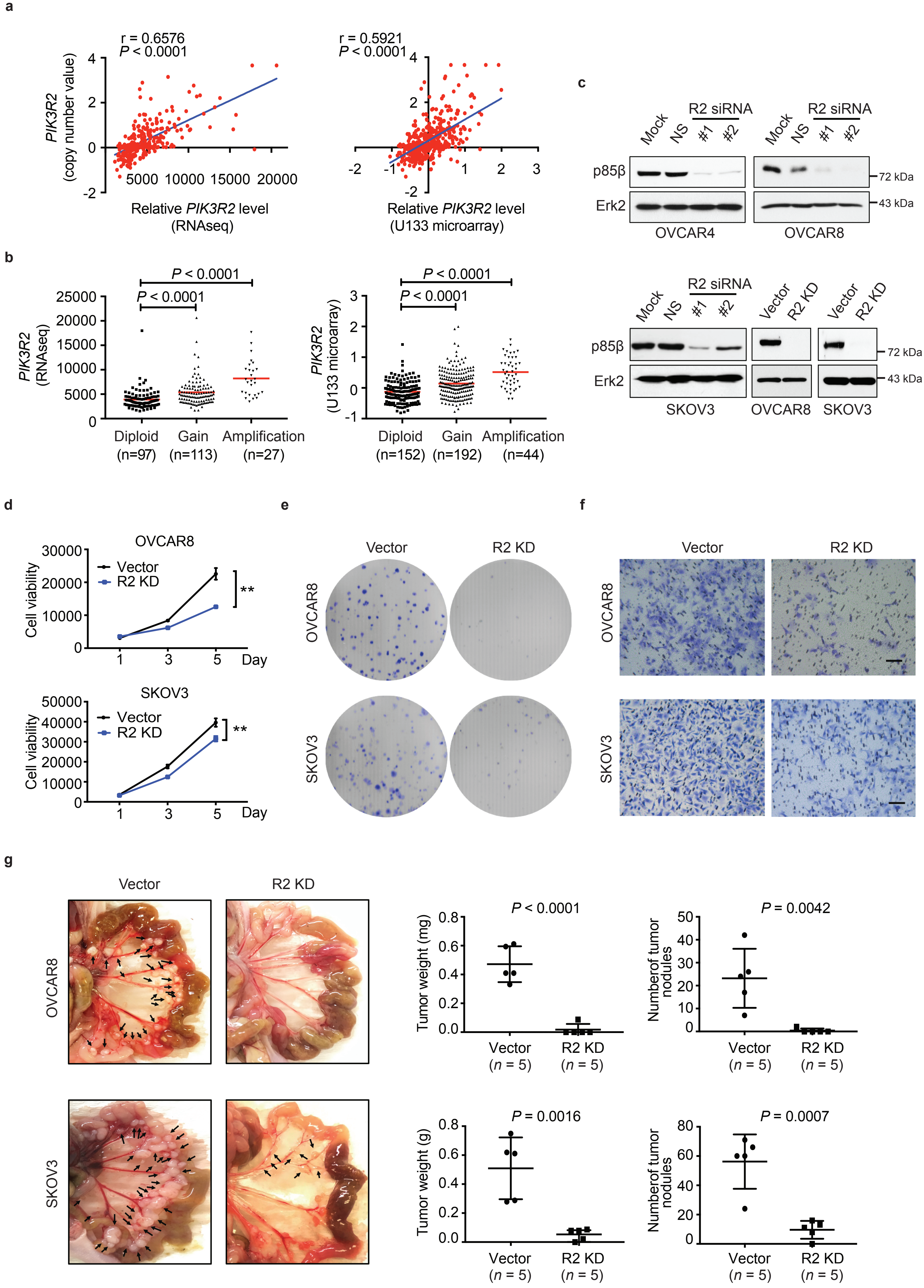

Supplementary Figure 1. p85β expression levels affect tumorigenicity of ovarian cancer cells. (**a**) Correlation of *PIK3R2* copy number values and relative *PIK3R2* mRNA levels in TCGA serous ovarian cancer samples by Pearson correlation analysis. *PIK3R2* mRNA levels were obtained from RNAseq (n = 301) or Affymetrix U133 microarray (n = 482). (**b**) *PIK3R2* mRNA level and *PIK3R2* copy number obtained from TCGA data; P value shown was analyzed by Mann-Whitney test. (**c**) Lysates of cells transfected with *PIK3R2* siRNA or stably expressing *PIK3R2* shRNA (R2 KD) were subjected to Western blotting for the protein levels of p85β. The experiment was repeated three times with independent lysates and results were reproducible. (**d-f**) Cells with or without stable *PIK3R2* knockdown (R2 KD) were subjected to (**d**) cell viability assay, (**e**) colony formation assay, and (**f**) cell invasion assay. Cell viability assay was done in triplicate and data are shown as mean ± SD. ** $P < 0.01$ using two-tailed *t*-test. Colony formation and invasion assays were performed three times and representative images are shown; Scale bars, 100 μm. (**g**) OVCAR8 or SKOV3 cells with or without stable *PIK3R2* knockdown were injected intraperitoneally into female nude mice for 6 weeks. Representative images show tumor nodules formed (arrows). Tumor weight and number of tumor nodules were measured with P values shown using two-tailed *t*-test. Data are shown as mean ± SD (n = 5). Source data are provided as a Source Data file.

Supplementary Figure 2

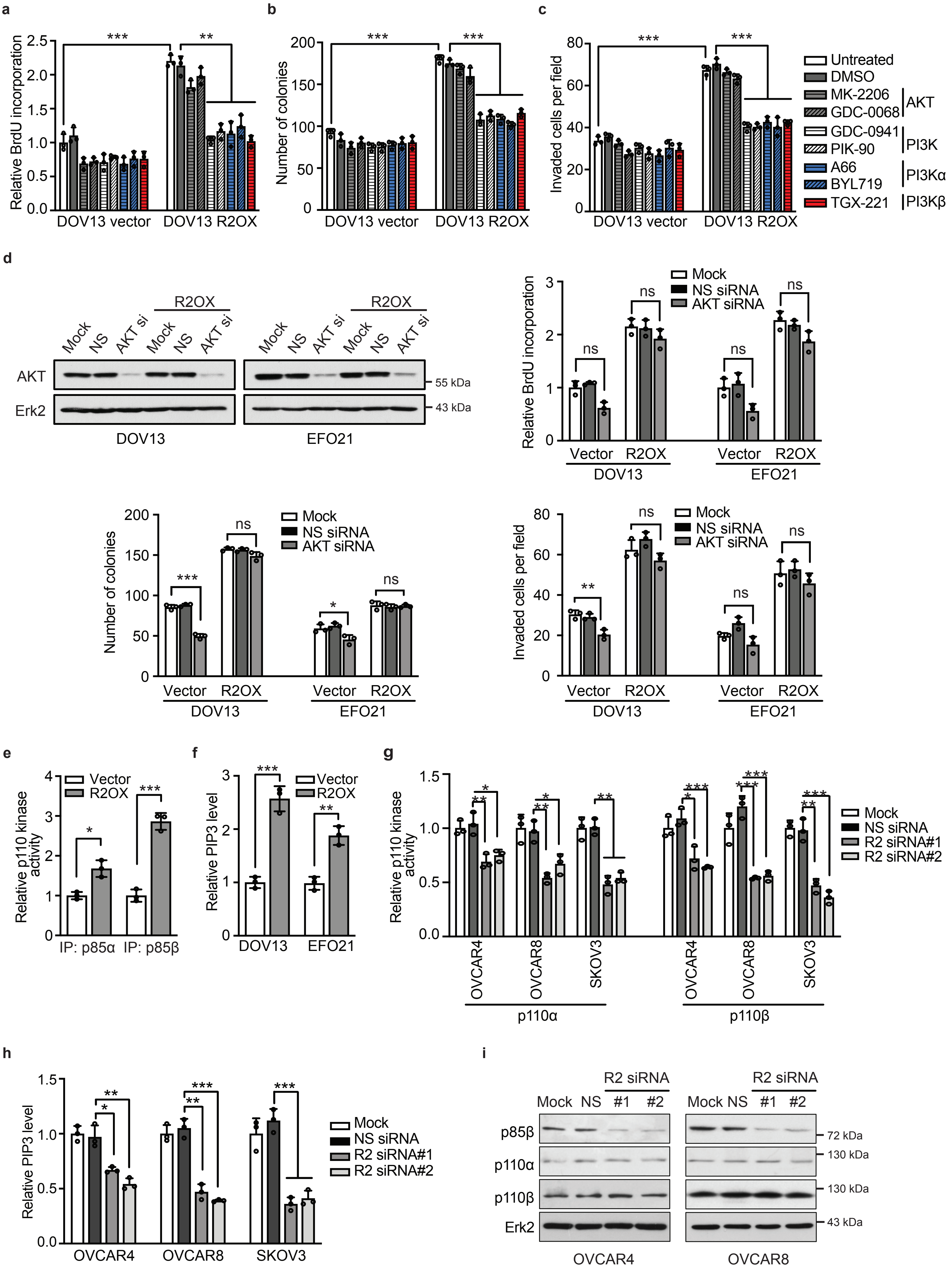

Supplementary Figure 2. p85β promotes p110 kinase activity but not p110 expression for the downstream oncogenic phenotypes. (**a-c**) DOV13 cells stably expressing *PIK3R2* (R2OX) or empty vector were treated with the indicated inhibitors and subjected to (**a**) BrdU cell proliferation assay, (**b**) colony formation assay, and (**c**) cell invasion assay. (**d**) DOV13 or EFO21 cells stably expressing *PIK3R2* (R2OX) or empty vector were transfected with siRNA and subjected to Western blotting, BrdU cell proliferation assay, colony formation assay and cell invasion assay. (**e**) Lysates of DOV13 cells expressing *PIK3R2* (R2OX) or vector control were immunoprecipitated with anti-p85α or anti-p85β antibody. Kinase activities of the bound p110 were then examined and the relative activities compared to vector control are shown. (**f**) Lipid from the indicated cells was collected, followed by PIP3 and PIP2 detection using ELISA kit. PIP3 level of each sample was normalized to that of PIP2 and the relative PIP3 levels compared to corresponding vector control are shown. (**g-i**) Cells were transfected with siRNA for 72 h before being harvested for (**g**) immunoprecipitation with anti-p110α or anti-p110β antibody prior to PI3-Kinase activity assay, (**h**) lipid ELISA assay to detect PIP3 and PIP2 levels, or (**i**) Western blotting for p85β, p110α and p110β protein levels. NS siRNA, non-specific siRNA. The assays were done in triplicate and representative data of three independent experiments are presented as mean ± SD. * $P < 0.05$; ** $P < 0.01$; *** $P < 0.001$ using two-tailed *t*-test. ns, not statistically significant. Source data are provided as a Source Data file.

Supplementary Figure 3

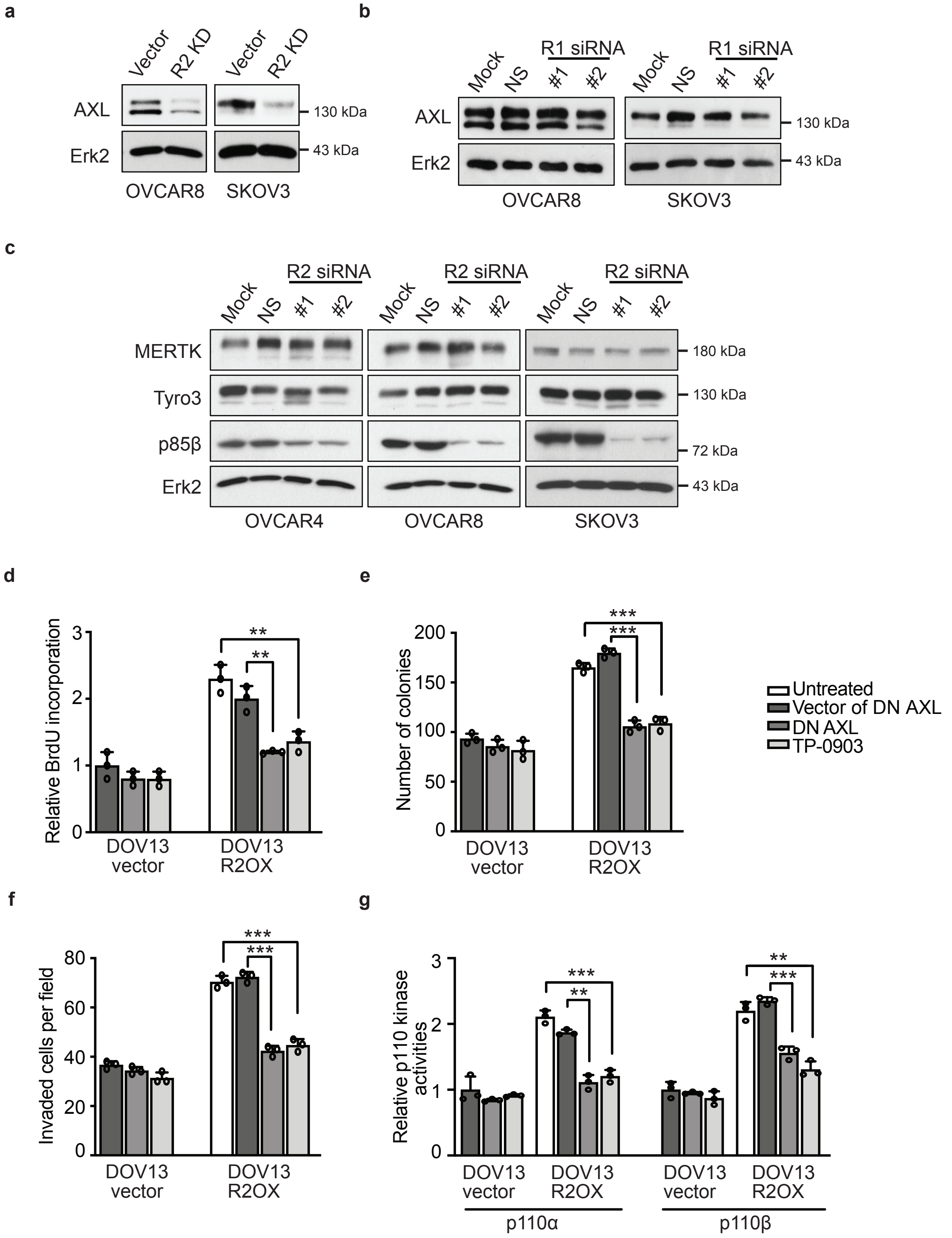

Supplementary Figure 3. p85β specifically increases AXL protein level to mediate its oncogenicity. (**a**) Protein levels of AXL and p85β in cells with or without stable *PIK3R2* knockdown (R2 KD). Erk2 was a loading control. (**b**) Expression of AXL in cells transfected with *PIK3R1* siRNA for 72 h. NS, non-specific siRNA. (**c**) Protein levels of MERTK and Tyro3 (the other two TAM members) in cells transfected with *PIK3R2* siRNA for 72 h were examined by Western blotting. The Western blotting experiments were repeated three times with independent lysates and results were reproducible. (**d-g**) DOV13 cells with or without stable *PIK3R2* overexpression (R2OX) were either treated with AXL inhibitor TP-0903 (0.5 μM), or transfected with dominant negative AXL (DN AXL) or its vector control. These cells were assayed for (**d**) BrdU cell proliferation, (**e**) colony formation, (**f**) cell invasion and (**g**) p110α or p110β PI3-kinase activity. Assays in **d-g** were done in triplicate and representative data of three independent experiments are presented as mean ± SD. ** $P < 0.01$; *** $P < 0.001$ using two-tailed *t*-test. Source data are provided as a Source Data file.

Supplementary Figure 4

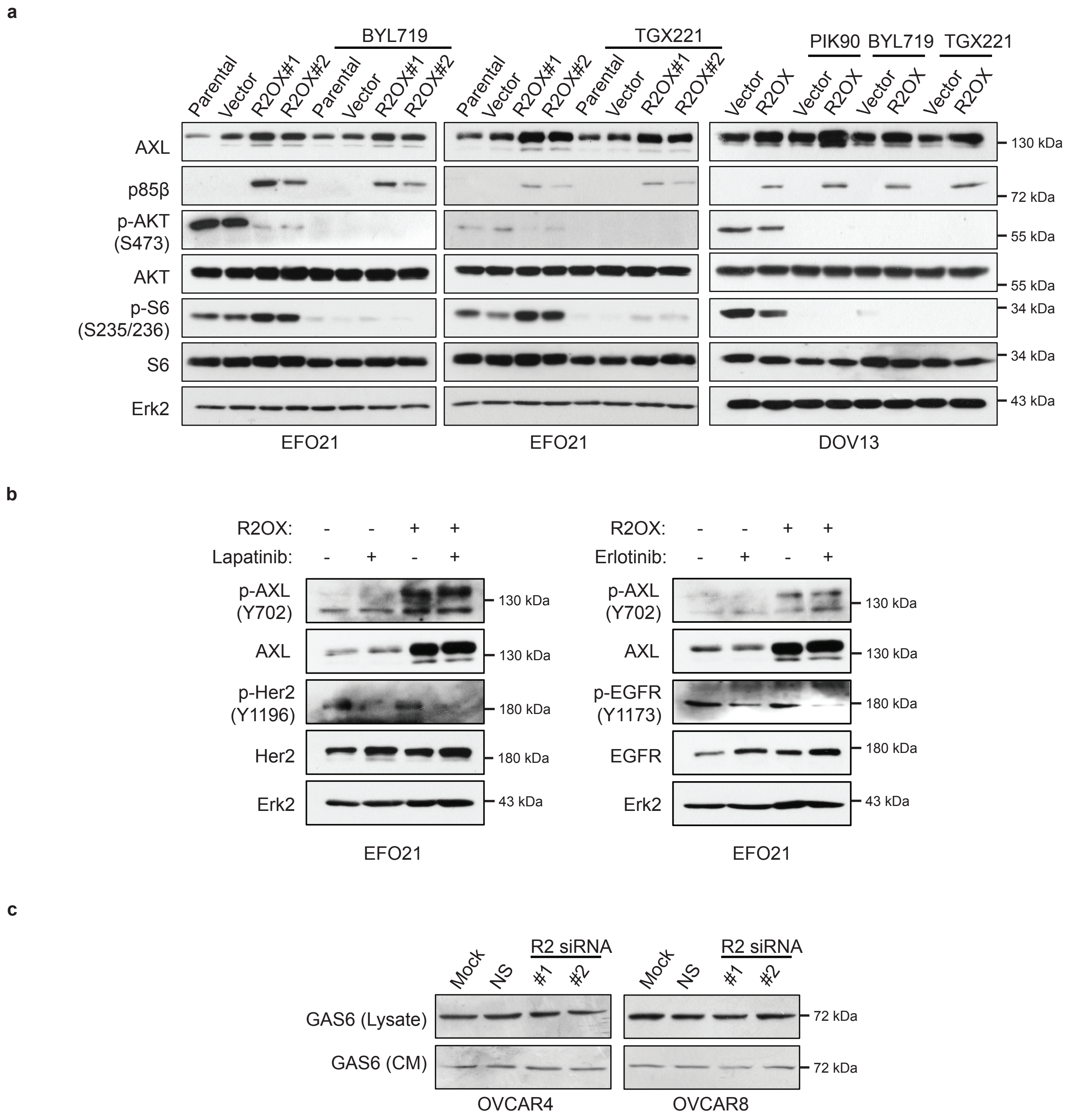

Supplementary Figure 4. The regulation of AXL by p85β does not involve p110, EGFR or HER2. (**a**) EFO21 or DOV13 cells stably expressing *PIK3R2* (R2OX) or vector control were treated with p110 inhibitors (PIK90: pan-p110 inhibitor, 10 μM; BYL719: p110α inhibitor, 2 μM; TGX221: p110β inhibitor, 10 μM) for 24 h. (**b**) EFO21 cells stably expressing *PIK3R2* (R2OX) or vector control were treated with lapatinib or erlotinib (5 μM) for 24 h. (**c**) Protein levels of GAS6 in cell lysates or conditioned medium (CM) of cells transfected with siRNA for 72 h. The experiments were repeated three times with independent lysates and results were reproducible. Source data are provided as a Source Data file.

Supplementary Figure 5

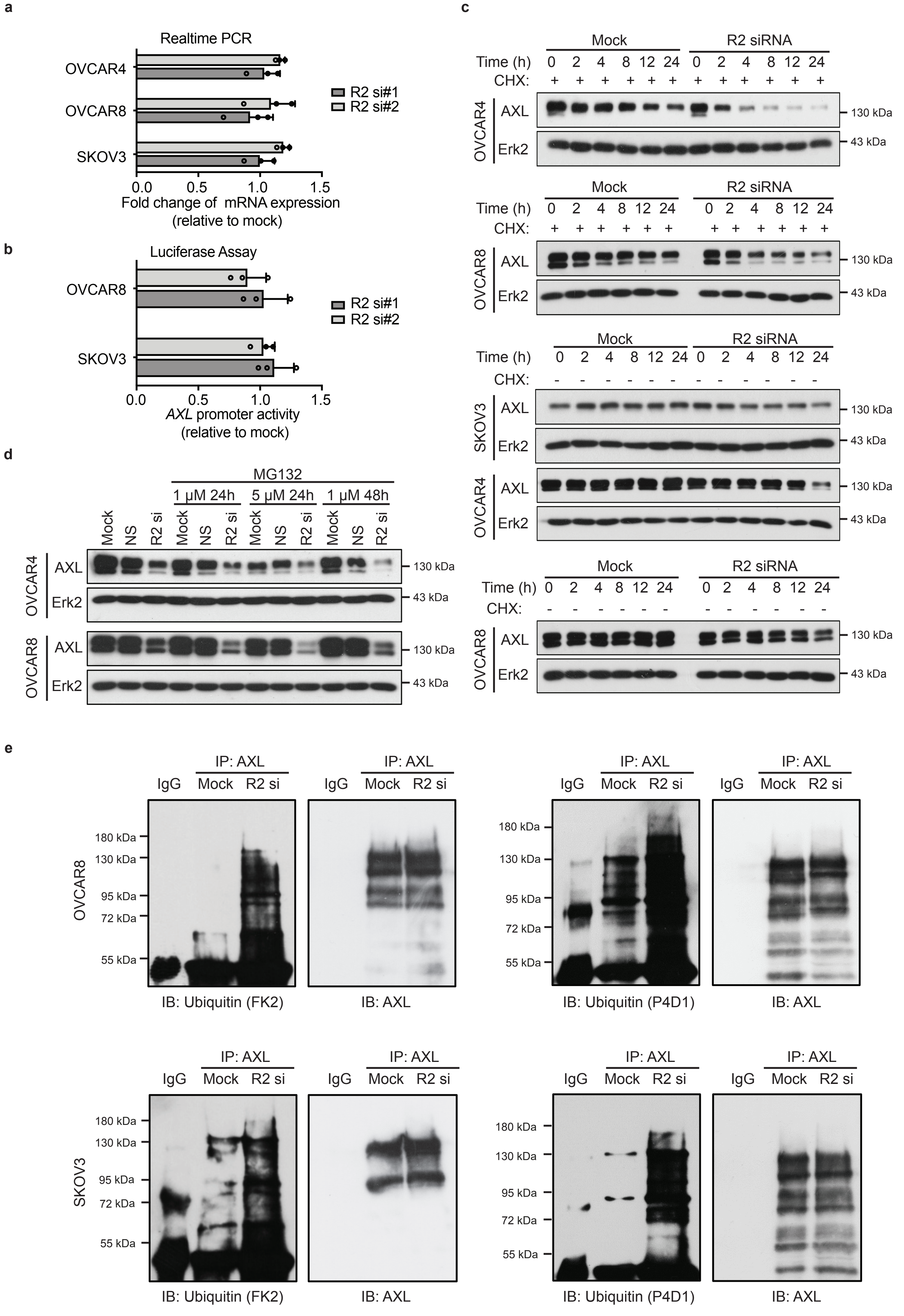

Supplementary Figure 5. p85β regulates AXL protein level post-transcriptionally through lysosomal but not proteasomal pathway. (**a**) Cells were transfected with *PIK3R2* siRNA (R2 si) for 72 h before being harvested for real-time PCR. (**b**) Cells were co-transfected with siRNA and human *AXL* promoter for 72 h prior to luciferase reporter assay. The relative values were calculated by comparing *PIK3R2* siRNA and the corresponding mock. Assays in **a-b** were done in triplicate and representative data of three independent experiments are presented as mean ± SD; no significant difference indicated by two-tailed *t*-test. (**c**) Cells transfected with siRNA for 36 h were then treated with or without 10 μg/mL cycloheximide (CHX, inhibitor of protein synthesis) for the indicated time course. (**d**) Cells transfected with *PIK3R2* siRNA for 36 h were treated with proteasome inhibitor MG132 at the indicated concentrations and durations. Protein levels of AXL and Erk2 (loading control) was examined. NS, non-specific siRNA. (**e**) AXL was immunoprecipitated (IP) from lysates of cells transfected with or without *PIK3R2* siRNA for 72 h. AXL protein levels were normalized prior to IP by using proportionally different amounts of input lysates. Independent sets of samples were subjected to Western blotting (IB) using anti-Ubiquitin antibodies (clones FK2 and P4D1). The membranes were stripped and re-probed with anti-AXL antibody. IP with rabbit IgG was negative control. The Western blotting experiments were repeated three times with independent lysates and results were reproducible. Source data are provided as a Source Data file.

Supplementary Figure 6

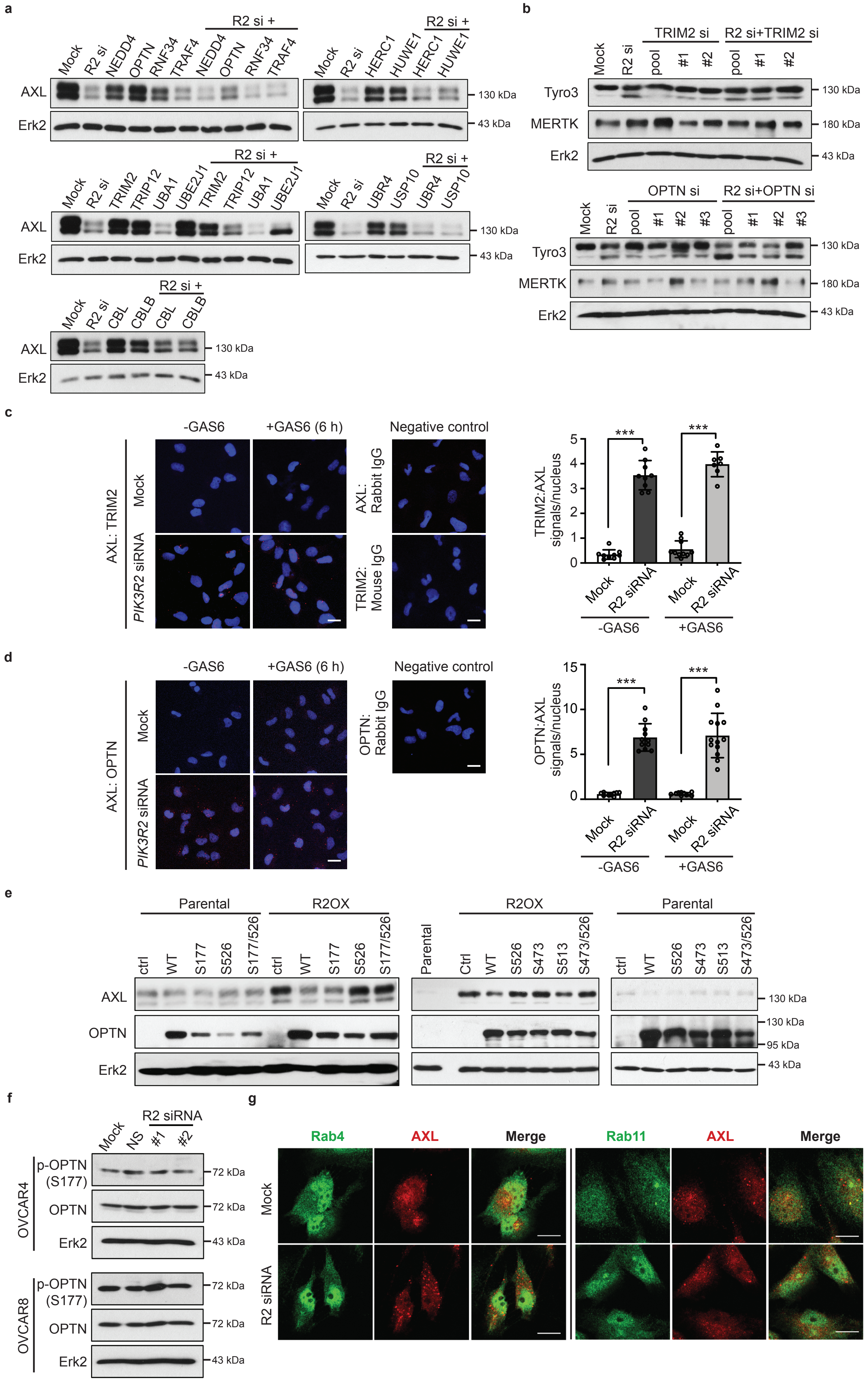

Supplementary Figure 6. TRIM2 and optineurin mediate the autophagy-lysosomal degradation of AXL regulated by p85β. (**a**) siRNA (SMARTpool) targeting 14 individual genes was transfected alone or in combination with *PIK3R2* siRNA into OVCAR8 cells for 72 h. (**b**) OVCAR8 cells were transfected with specific siRNAs of *OPTN* or *TRIM2* (pool represents RNAi SMARTpool, whereas the numbers indicate the different individual sequences) alone or in combination with *PIK3R2* siRNA for 72 h. Cell lysates were examined for the protein levels of Tyro3 and MERTK with Erk2 as loading control. (**c-d**) OVCAR8 cells transfected with non-specific or *PIK3R2* siRNA were serum-starved for 24 h prior to stimulation with Gas6 (500 ng/ml) for 6 h. Cells were subjected to proximity ligation assay with the indicated antibodies. Negative controls with one of the antibodies replaced by IgG were included. Representative images are shown (left). Scale bars, 20 μm. The number of signals per nucleus was counted in ≥8 fields and data represent mean±SD (right). *** $P < 0.001$ using two-tailed *t*-test. (**e**) EFO21 cells stably expressing *PIK3R2* (R2OX) or vector were transfected with expression plasmids of *OPTN* or mutants (S177A, S473A or S513A) for 72 h. Protein levels of AXL, OPTN and Erk2 was examined. (**f**) OVCAR4 or OVCAR8 cells were transfected with siRNA for 72 h before being harvested for Western blotting. (**g**) OVCAR8 transfected with *PIK3R2* siRNA for 48 h was subjected to immunofluorescence staining using the indicated antibodies followed by confocal microscopy. Representative images are shown. Scale bars, 20 μm. The experiments were repeated three times with independent samples and results were reproducible. Source data are provided as a Source Data file.

Supplementary Figure 7

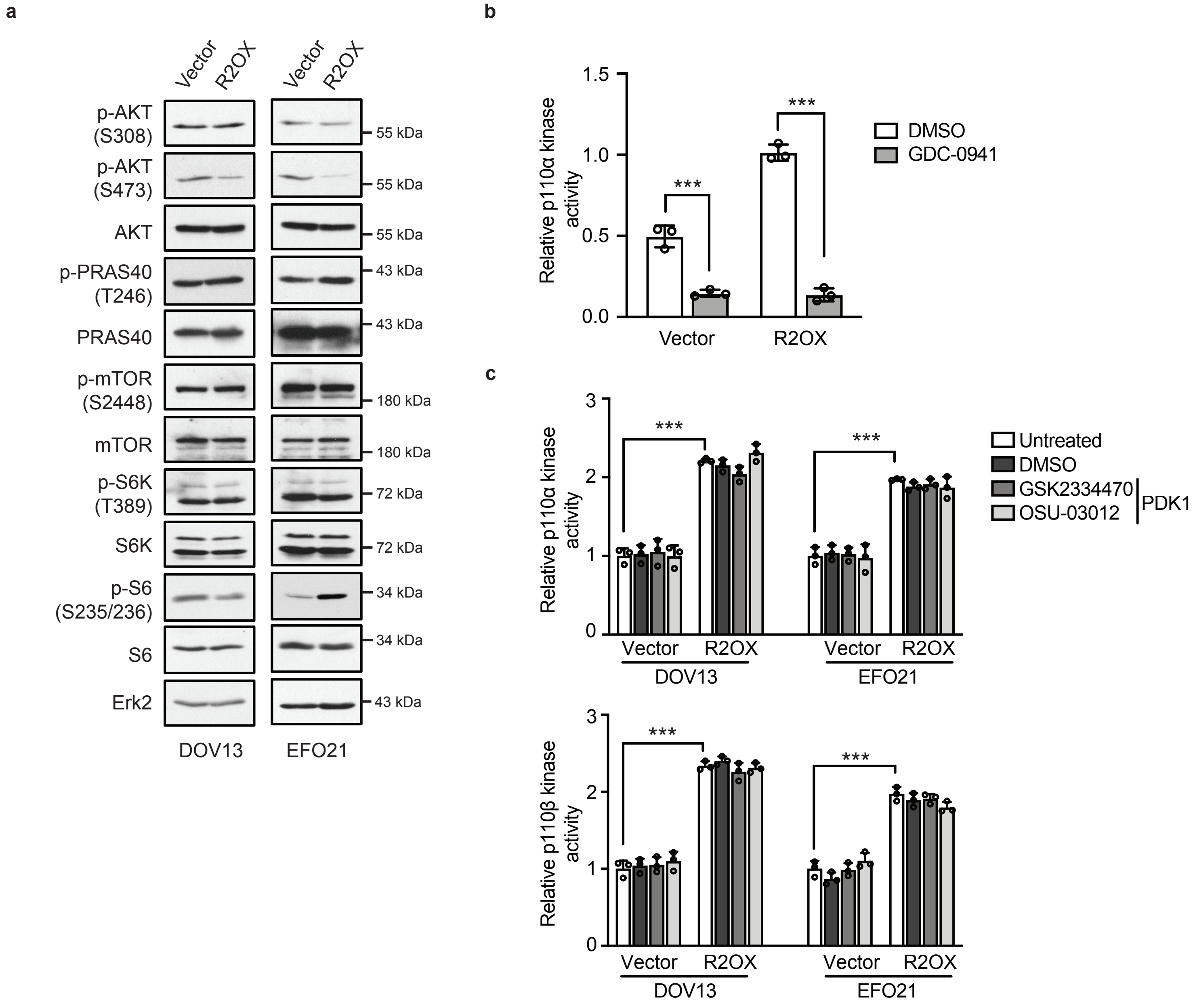

Supplementary Figure 7. PDK1 does not mediate p85β-induced p110 kinase activity. (**a**) DOV13 or EFO21 cells stably expressing *PIK3R2* (R2OX) or vector were harvested for Western blotting. The experiment was repeated three times with independent lysates and results were reproducible. (**b**) EFO21 cells stably expressing *PIK3R2* (R2OX) or vector were treated with pan p110 inhibitor GDC-0941 (10 μM) for 48 h prior to immunoprecipitation with anti-p110α antibody for PI3-Kinase activity assay. (**c**) DOV13 or EFO21 cells stably expressing *PIK3R2* (R2OX) or vector were treated with PDK1 inhibitor GSK2334470 (2 μM) or OSU-03012 (5 μM) for 24 h. Cell lysates were immunoprecipitated using anti-p110α (left) or anti-p110β (right) antibody prior to PI3-Kinase activity assay. Assays were done in triplicate and representative data of three independent experiments are presented as mean ± SD. *** $P < 0.001$ using two-tailed *t*-test. Source data are provided as a Source Data file.

Supplementary Figure 8

a

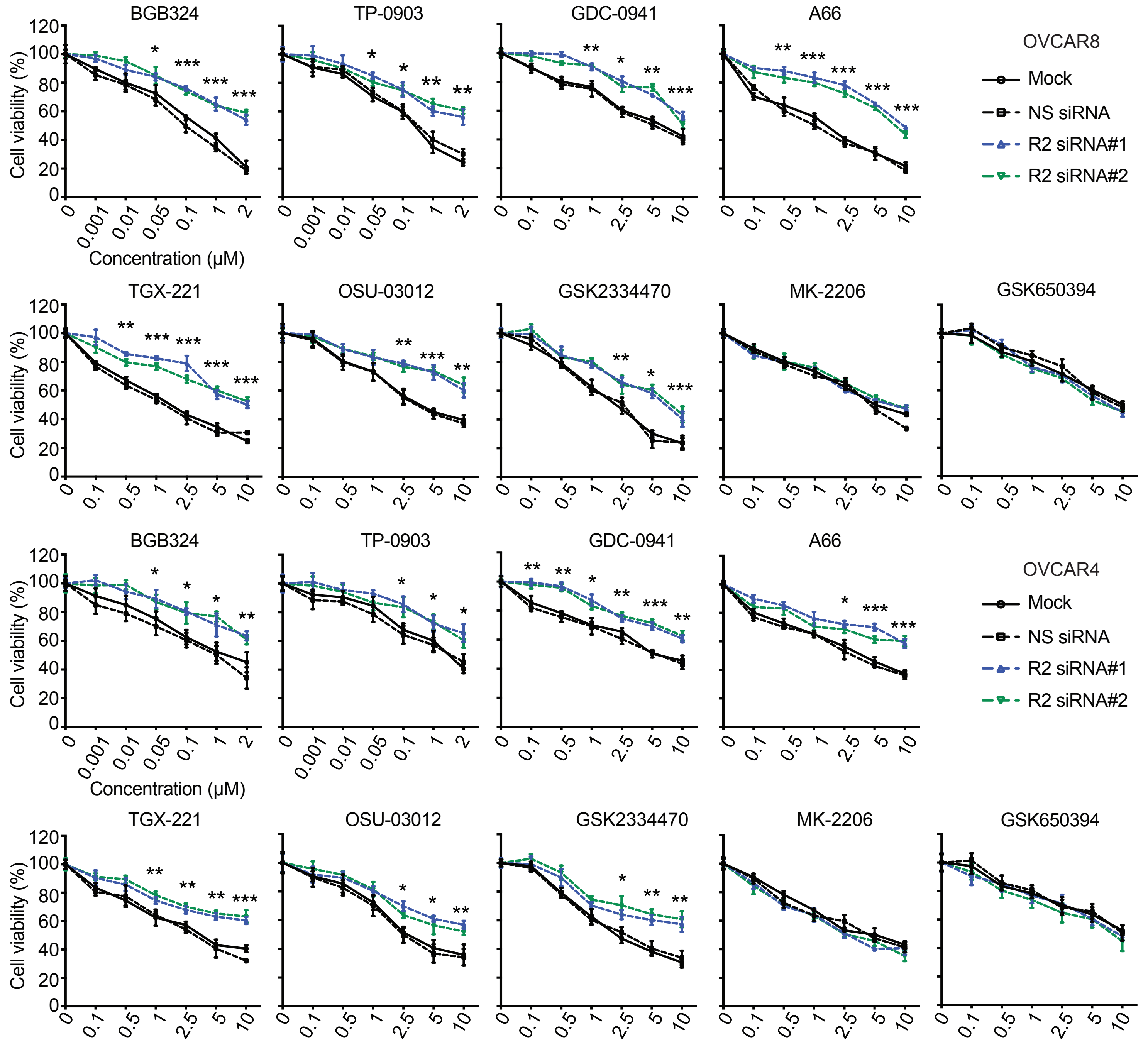

b

		OVCAR8				OVCAR4			
		Mock	NS	R2si#1	R2si#2	Mock	NS	R2si#1	R2si#2
AXL	BGB324	0.21	0.12	1.88	2.08	0.98	0.59	2.81	2.93
	TP-0903	0.21	0.34	1.79	2.22	1.18	1.16	3.11	2.73
p110	GDC-0941	4.79	4.31	12.20	11.19	4.83	4.27	11.73	12.61
p110α	A66	1.38	1.16	8.74	6.89	3.20	2.78	9.84	7.90
p110β	TGX-221	1.65	1.44	7.89	6.96	3.19	2.77	8.51	10.05
PDK1	OSU-03012	3.82	3.56	12.38	13.32	3.26	2.85	8.31	6.73
	GSK2334470	2.08	2.05	5.73	6.15	2.46	2.69	7.18	9.41
AKT	MK-2206	3.79	3.63	2.77	2.78	4.69	3.98	5.02	5.68
SGK1/2	GSK650394	7.82	7.91	6.97	5.82	7.54	7.86	6.42	5.73

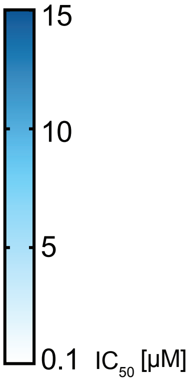

Supplementary Figure 8. Cells with *PIK3R2* depletion are less sensitive to inhibitors of AXL/PDK1 signaling. (**a**) OVCAR8 and OVCAR4 3D spheriods transfected with siRNA were treated with indicated inhibitors for 72 h. Dose-response curves of each inhibitor are shown. NS siRNA, non-specific siRNA. Data shown represent mean ± SD (n = 3 biologically independent samples). * $P < 0.05$, ** $P < 0.01$, *** $P < 0.001$ by two-tailed *t*-test. (**b**) Heatmap illustrating corresponding IC50 values of each inhibitor. Source data are provided as a Source Data file.

Supplementary Figure 9

a

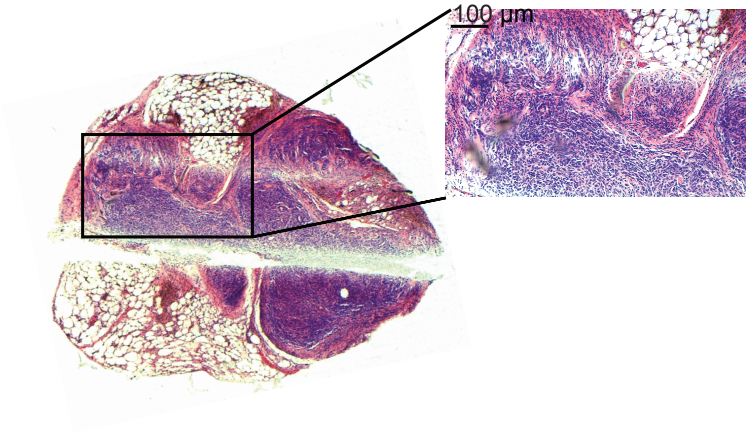

b

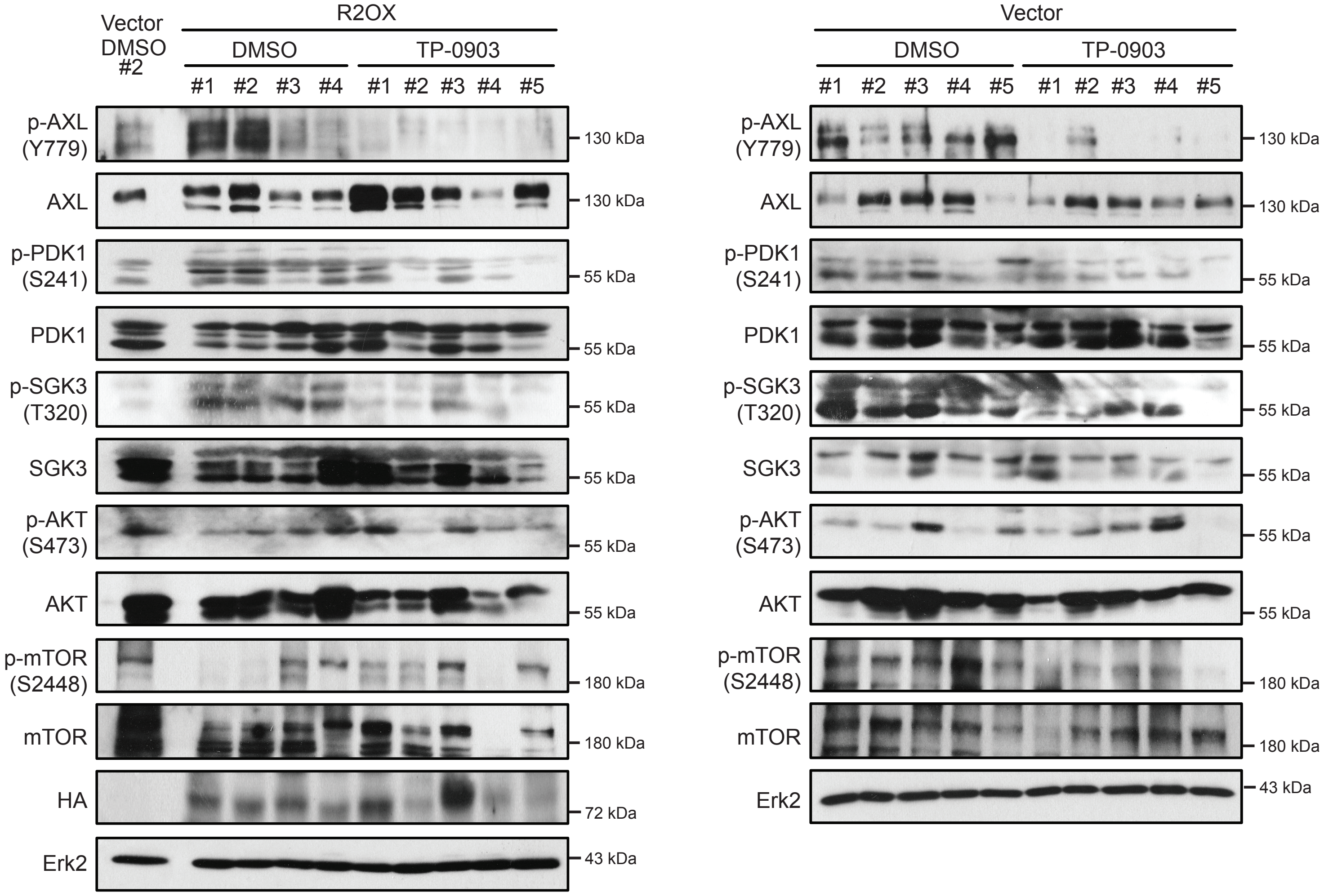

Supplementary Figure 9. Xenograft tumors stably expressing *PIK3R2* are sensitive to AXL inhibitor. Mice were injected i.p. with DOV13 cells stably expressing *PIK3R2* (R2OX) or vector and then treated with TP-0903 or vehicle control DMSO. Tumor nodules were collected at experiment endpoint. (**a**) H&E staining of an xenograft tumor section derived from R2OX. Scale bar, 100 µm. (**b**) Protein was extracted from the tumor nodules to examine the levels of the indicated signaling molecules and HA tag (representing *PIK3R2* overexpression) with Erk2 as a loading control. Source data are provided as a Source Data file.

Supplementary Table 1. Proteins which showed significant changes in phosphorylation after *PIK3R2* knockdown

	Gene names	Amino acid	Positions within proteins	Log2 Intensity NS siRNA_1	Log2 Intensity NS siRNA_2	Log2 Intensity R2 siRNA_1	Log2 Intensity R2 siRNA_2	FC of means_R2si Vs Nssi	*P* value (two-sided t-test)
ABLIM1	ABLIM1	S	452	23.44746	23.60998	23.93094	24.01078	0.44214	0.03
		S	455	23.44746	23.60998	23.93094	24.01078	0.44214	0.03
ADAR	ADAR	S	823	24.21911	23.17963	20.45368	19.98204	-3.48151	0.02
AHNAK	AHNAK	S	5749	25.68137	25.12116	20.41278	20.10015	-5.1448	0.003
APC	APC	S	2449	21.80356	22.00693	20.97839	20.93654	-0.94778	0.01
ATAT1	ATAT1	S	315	20.95352	19.52285	23.2359	23.00029	2.87991	0.05
CFL1	CFL1	S	3	28.66207	28.8744	29.56313	29.83482	0.93074	0.03
CFL2	CFL2	S	3	22.2064	22.01529	23.25782	23.58761	1.31187	0.02
CSNK1E	CSNK1E	S	363	26.40855	26.45387	21.03573	19.84178	-5.992455	0.009
CTTNBP2NL	CTTNBP2NL	S	443	21.17848	20.8659	23.6479	22.61816	2.11084	0.05
DCLK1	DCLK1	S	332	22.54	21.96399	23.33824	23.51521	1.17473	0.05
DLG5	DLG5	S	1254	24.25097	23.00161	20.6058	19.77791	-3.434435	0.04
DSTN	DSTN	S	3	26.57903	26.83629	28.00908	27.63159	1.112675	0.03
DSTYK	DSTYK	S	928	20.79251	19.46345	22.81705	22.88729	2.72419	0.05
EIF5B	EIF5B	S	186	24.15167	23.06406	19.6758	19.57514	-3.982395	0.01
		S	190	24.15167	23.06406	21.19455	20.71251	-2.654335	0.04
FKBP15	FKBP15	S	1164	23.25078	22.6988	20.92945	20.28261	-2.36876	0.03
GNG12	GNG12	S	3	21.52094	20.45987	24.54699	24.46407	3.515125	0.02
HERC1	HERC1	S	1428	24.41235	23.144	20.37183	20.72652	-3.229	0.03
HIST1H1C	HIST1H1C	S	36	25.15738	25.12719	24.69954	24.69376	-0.445635	0.001
HIST1H1E; HIST1H1D	HIST1H1E; HIST1H1D	S	36;37	25.15738	25.12719	24.69954	24.69376	-0.445635	0.001
HMGA1	HMGA1	S	99	19.96972	20.65022	29.11197	27.67755	8.08479	0.009

**HUWE1**	**HUWE1**	S	2595	24.45262	24.25516	25.04439	24.82653	0.58157	0.05
		S	2593	21.60876	21.94206	23.30689	22.76549	1.26078	0.05
**IGF1R**	**IGF1R**	T	1366	20.93727	20.94228	21.26316	21.20359	0.2936	0.01
**ITSN2**	**ITSN2**	S	884	22.18966	22.10843	23.85472	23.17796	1.367295	0.05
		S	889	22.18966	22.10843	23.85472	23.17796	1.367295	0.05
**KAT6A**	**KAT6A**	S	1089	23.9157	22.73583	20.32651	20.25791	-3.033555	0.03
**KIAA1551**	**KIAA1551**	S	1744	24.3965	24.88688	20.80123	20.66403	-3.90906	0.004
		S	1740	24.3965	24.88688	21.24468	20.62879	-3.704955	0.01
**KIF13B**	**KIF13B**	S	1778	23.4545	23.36279	22.95036	22.94442	-0.461255	0.009
**KIRREL3**	**KIRREL3**	S	596	21.23372	21.38068	21.83584	21.94971	0.585575	0.02
**KRT8**	**KRT8**	S	2;2;23	27.8769	27.99895	28.47796	28.43468	0.518395	0.01
		S	457;457;417	21.05729	19.88702	24.53558	24.09555	3.84341	0.02
**LARP1**	**LARP1**	S	548	29.45468	29.52086	29.76419	29.71787	0.25326	0.02
**MFSD6**	**MFSD6**	T	10	21.86685	21.539	25.17512	24.65753	3.2134	0.009
**MICALL1**	**MICALL1**	S	578	22.64438	22.49826	21.47303	21.82501	-0.9223	0.04
**MMTAG2**	**MMTAG2**	S	217	27.09275	25.59471	21.34757	20.84236	-5.248765	0.02
**NAP1L1**	**NAP1L1**	S	10	21.72028	20.81639	23.77766	23.6753	2.458145	0.03
**NEDD4**	**NEDD4**	S	737	20.86494	20.73385	23.26785	22.62417	2.146615	0.02
**OPTN**	**OPTN**	S	526	22.2626	21.17766	27.84146	26.39689	5.399045	0.02
**OSTM1**	**OSTM1**	S	322	25.65657	25.80336	25.21423	25.11398	-0.56586	0.02
**PHLDA1**	**PHLDA1**	S	398	21.90292	19.49848	27.52305	26.92116	6.521405	0.03
**PKP2**	**PKP2**	S	135	22.27714	22.5503	20.80022	20.07244	-1.97739	0.03
**PNN**	**PNN**	S	695	21.54316	20.05916	23.90545	23.78207	3.0426	0.05
**PPIG**	**PPIG**	S	375	25.719	24.94351	21.77045	20.07086	-4.4106	0.04
**PPM1A**	**PPM1A**	T	376	21.58636	21.46976	20.86435	20.92789	-0.63194	0.01
**PRKD2**	**PRKD2**	S	200	24.14608	22.89165	20.99894	20.71091	-2.66394	0.05
**REEP4**	**REEP4**	S	202	21.42901	20.65156	22.69543	23.01427	1.814565	0.04
**RNF34**	**RNF34**	S	256	21.3093	21.29424	23.26714	23.07407	1.868835	0.002

**SAFB2; SAFB**	**SAFB2; SAFB**	S	444;443	24.92311	24.61762	25.46865	25.44787	0.687895	0.04
**SASH1**	**SASH1**	S	355	24.42739	23.88762	20.36844	20.95804	-3.494265	0.01
**SCAMP2**	**SCAMP2**	S	319	26.48971	26.5819	26.11967	26.26067	-0.345635	0.05
**SERBP1**	**SERBP1**	S	197	24.77087	23.50506	20.46814	19.8335	-3.987145	0.03
**SFSWAP**	**SFSWAP**	S	834	21.37094	19.60831	24.24351	24.12289	3.693575	0.05
**SLCO4A1**	**SLCO4A1**	S	43	21.17774	21.67126	24.18715	23.80134	2.569745	0.01
**SNIP1**	**SNIP1**	S	52	21.17397	20.34503	24.04519	23.16185	2.84402	0.04
		S	54	21.16656	20.03648	24.04519	23.16185	3.002	0.05
**SRRM1**	**SRRM1**	S	749	24.82929	24.00505	21.41352	19.66714	-3.87684	0.05
**SRRM2**	**SRRM2**	T	1986	27.56827	26.79971	20.23033	19.93121	-7.10322	0.003
		S	1923	27.37426	25.87968	20.83755	20.06837	-6.17401	0.01
		S	1905	25.37637	23.92751	20.43452	19.91663	-4.476365	0.02
		S	914	22.04416	19.66737	26.2236	25.51875	5.01541	0.05
		S	838	20.79564	20.12436	25.33385	23.85994	4.136895	0.03
**STARD3**	**STARD3**	S	213	21.23551	21.32427	23.83807	23.07178	2.175035	0.03
**STK11IP**	**STK11IP**	S	403	23.34854	23.01772	20.79715	20.24478	-2.662165	0.01
**TBC1D5**	**TBC1D5**	S	522	22.11194	22.17103	23.15731	22.88826	0.8813	0.02
**THOC1**	**THOC1**	S	2	23.87155	23.78526	24.04618	24.07431	0.23184	0.03
**THRAP3**	**THRAP3**	S	408	24.38179	23.0085	20.8256	20.49561	-3.03454	0.05
**TMEM109**	**TMEM109**	S	239	27.59989	26.77819	21.59607	19.61347	-6.58427	0.02
**TNS3**	**TNS3**	S	1149	24.62238	22.91161	20.35936	19.6074	-3.783615	0.05
**TP53BP1**	**TP53BP1**	S	1481	20.51375	19.61879	23.6685	23.46351	3.499735	0.01
**TPR**	**TPR**	S	2073	24.3674	24.35118	25.26882	25.00243	0.776335	0.02
**TRAF2**	**TRAF2**	T	7	20.76862	20.20822	26.50786	25.91061	5.720815	0.005
**TRIM2**	**TRIM2**	S	443	20.98229	20.89375	22.86505	23.30786	2.148435	0.01
**TRIP12**	**TRIP12**	S	1376	21.52043	21.26218	22.45981	22.32378	1.00049	0.02
		S	310	21.5781	20.44471	24.08148	23.85339	2.95603	0.03
**UBA1**	**UBA1**	S	4	20.68961	19.13619	25.68806	25.92653	5.894395	0.01

**UBE2J1**	**UBE2J1**	S	266	20.83912	20.59307	24.47519	23.56929	3.306145	0.01
**UBR4**	**UBR4**	S	178	22.06854	22.27301	23.3948	23.30618	1.179715	0.008
**USP10**	**USP10**	T	208	21.07599	21.38574	24.06308	23.62901	2.61518	0.01
**VGLL4**	**VGLL4**	S	103	21.76969	20.40556	25.42618	24.15908	3.705005	0.05
		S	103	21.66418	20.77085	25.96869	25.06936	4.30151	0.02
		S	101	20.57965	19.76418	25.42618	24.15908	4.620715	0.02
**XPR1**	**XPR1**	T	690	21.11781	21.12237	21.63542	21.57348	0.48436	0.004
**ZC3H13**	**ZC3H13**	S	109	21.5455	21.3618	23.80637	23.67616	2.287615	0.002
		S	207	22.15102	21.27376	24.90871	24.44462	2.964275	0.02
		S	209	20.95453	20.06836	24.90871	24.44462	4.16522	0.01
**ZNF318**	**ZNF318**	S	69	22.07458	20.60006	26.58735	26.28761	5.10016	0.02
		S	71	21.22156	20.40969	26.58735	26.28761	5.621855	0.005

Supplementary Table 2. Sequences of siRNA, shRNA and siRNA SmartPool

	Gene	Sequence#1	Sequence#2
siRNA	*PIK3R2*	GCGCCCAGCUUAAGGUCUA	GGAAAGGCGGGAACAAUAA
	TRIM2	UCAAAGUCUAUCGAUACUUACAGTA	AUUUCUUCAUCACAAACCUGAUGGA
	NEDD4	CAUGUUUACAGAUUAAGCUAAUUTC	GGACUUGCCACCUUAUGAAUCAUTT
	OPTN	#1: UGGAAAGCAUGCUAUCAGAAAUCAA<br>#3: GCUAAUGAAGAAGAGACUUCAAGAA	#2: GCAGAAGGGUCAGUAAAAGAAAUCA
	AKT1	ACAAGGACGGGCACAUUAA	
	AKT2	ACACAAGGUACUUCGAUGA	
	AKT3	GCACACACUCUAACUGAAA	
shRNA	*PIK3R2*	CAGATGAAGCGTACTGCAATT	
siRNA SmartPool	*CBL*	#1: AAUCAACUCUGAACGGAAA; #2: GACAAUCCCUCACAAUAAA<br>#3: UAGCCCACCUUAUAUCUUA; #4: GGAGACACAUUUCGGAUUA	
	CBLB	#1: GAACAUCACAGGACUAUGA; #2: GUACUGGUCCGUUAGCAAA<br>#3: GGUCGAAUUUUGGGUAUUA; #4: UAUCAGCAUUUACGACUUA	
	HERC1	#1: GCACCGACCUUAUUGUGUA; #2: UAGAUUAGCUUCUGAGUUG<br>#3: CCACAGGUCCUAUUACUAA; #4: GAACAAAGGAACCACUUGA	
	HUWE1	#1: GCUUUGGGCUGGCCUAAUA; #2: GCAGUUGGCGGCUUUCUUA<br>#3: GAGCCCAGAUGACUAAGUA; #4: UAACAUCAAUUGUCCACUU	
	NEDD4	#1: GGAGGGAACAUACAAAGUA; #2: GAUCACAAUUCCAGAACGA<br>#3: GAACUAGAGCUUCUUAUGU; #4: CCAAUGAUCUAGGGCCUUU	
	OPTN	#1: GGGCUCAGAUGGAAGUUUA; #2: CCAUGAAGCUAAAUAAUCA<br>#3: CUUCGAACAUGAGGAGUUA; #4: CUAAUGGCCUUGAGUCAUG	
	RNF34	#1: UCUGAGAAAUAUACCCAUA; #2: CGGCACAGGUACAAAGUGA<br>#3: GGCCCAACAUAGUUUGUAA; #4: GCUUAUGGAUGGAGACCAA	
	TRAF2	#1: CCGCAUACCCGCCAUCUUC; #2: GCAGGUACGGCUACAAGAU<br>#3: CGACGUGACUUCAUCCUCU; #4: GGACCAAGCUGGAAGCCAA	
	TRIM2	#1: GUAUAUGCCUGGAACGGUA; #2: CAACCAAUGUGUGCAGAUA<br>#3: GGUCAACUAUGGCCUCAAA; #4: GCAAGAGUGUGCUGCUUAU	
	TRIP12	#1: GAACACAGAUGGUGCGAUA; #2: GACAAAGACUCAUACAAUA<br>#3: GCUCAUAUCGCAAAGGUUA; #4: GGUAGUGACUCCACCCAUU	
	UBA1	#1: GCGUGGAGAUCGCUAAGAA; #2: CCUUAUACCUUUAGCAUCU<br>#3: CCACAUAUCCGGGUGACAA; #4: GAAGUCAAAUCUGAAUCGA	

	UBE2J1	#1: GCUCUUAUAUUCCGACGAA; #2: GAGUAUAAGGACAGCAUUA<br>#3: GAUGUCCUGUUGCCUUUAA; #4: GCCAUAGGUUCUCUAGAUU
	UBR4	#1: GGGAACACCCUGACGUAAA; #2: UCAUGAAGCCUGUUCGAAA<br>#3: CUACGAAGCUGCCGACAAA; #4: UGAACAAAUUUGCCGAUAA
	USP10	#1: UGAGUUUGGUGUCGAUGAA; #2: GAUAAAAUCGUGAGGGAUA<br>#3: GGAAAAUGAUGGUGUCUCA; #4: AAGCUUCUCUCACCAAGUA

Supplementary Table 3. Primer sequences for realtime PCR

Gene	Forward	Reverse
AXL	CGAAAGAAGGAGACCCGTTATG	ATAGAGGAGGAAGCTGTGTAGG
GAPDH	TCCATGACAACTTTGGTATCGTG	ACAGTCTTCTGGGTGGCAGTG

Supplementary Table 4. Antibodies used in this study

Antibody	Protein	Supplier	Catalog #	WB	IF	IHC	PLA	IP
Anti-p85β	p85β	Abcam	ab28356	1:1000		1:25		
		Santa Cruz	sc-56934				1:35	
Anti-AXL	AXL	Cell Signaling	8661	1:2000	1:160	1:40	1:160	
		Santa Cruz	sc-166268		1:33			2μg antibody/1mg protein input
		R&D	AF154	1:1000				
Anti-phospho-AXL (Y779)	AXL pY779	R&D	AF2228	1:400				
Anti-phospho-AXL (Y702)	AXL pY702	Cell Signaling	5724	1:1000				
Anti-Tyro3	Tyro3	Cell Signaling	5585	1:1000				
Anti-MERTK	MERTK	Santa Cruz	sc-365499	1:800				
Anti-HA	HA	Biolegend	901501	1:2000				
Anti-phospho-AKT (S473)	AKT pS473	Cell Signaling	9271	1:1000				
Anti-phospho-AKT (T308)	AKT pT308	Santa Cruz	sc-271966	1:1000				
Anti-AKT	AKT	Cell Signaling	4691	1:3000				
Ani-ERK2	ERK2	Santa Cruz	sc-154	1:5000				
Anti-ATG5	ATG5	Santa Cruz	sc-133158		1:30			
Anti-ATG12	ATG12	Cell Signaling	2010		1:60			
Anti-Ubiquitin (FK2)	Ubiquitin	Enzo	BML-PW8810	1:500				
Anti-Ubiquitin (P4D1)	Ubiquitin	Santa Cruz	sc-0817	1:400				
Anti-TRIM2	TRIM2	Abcam	ab3942	1:700				
		Proteintech	20356-1-AP	1:2000			1:40	2μg antibody/1mg protein input
Anti-OPTN	OPTN	Sigma	HPA003360	1:250				
		Santa Cruz	sc-271549				1:35	2μg antibody/1mg protein input
Anti-phospho-OPTN (S177)	OPTN pS177	Cell Signaling	57548	1:1000				
Anti-NEDD4	NEDD4	Cell Signaling	2740	1:1000				
Anti-GAS6	GAS6	R&D	AF885	1:1000				
Anti-S6	S6	Cell Signaling	2317	1:1000				
Anti-phsopho-S6 (Ser235/236)	S6 pS235/236	Cell Signaling	2211	1:4000				
Anti-Rab4	Rab4	Cell Signaling	2167		1:50			
Anti-Rab7	Rab7	Santa Cruz	sc-376362		1:80			
Anti-Rab11	Rab11	Cell Signaling	5589		1:50			

Anti-LC3B	LC3B	Abcam	ab51520	1:3000	1:500				
Anti-p110α	p110α	Cell Signaling	4255	1:750					
Anti-p110β	p110β	Santa Cruz	sc-376412	1:100					
Anti-phosphotyrosine	p-Tyr	Millipore	05-321X	1:1000					
Anti-phospho-PDK1 (S241)	PDK1 pS241	Cell Signaling	3061	1:1000					
Anti-PDK1	PDK1	Cell Signaling	3062	1:800					
Anti-phospho-SGK3 (T320)	SGK pT320	Cell Signaling	5642	1:1000					
Anti-SGK3	SGK3	Cell Signaling	8156	1:1000					
Anti-phospho-NDRG1 (T346)	NDRG1 pT346	Cell Signaling	3217	1:1000					
Anti-NDRG1	NDRG1	Abcam	ab37897	1:1000					
Anti-phospho-SGK1 (S78)	SGK1 pS78	Cell Signaling	5599	1:1000					
Anti-SGK1	SGK1	Cell Signaling	12103	1:1000					
Anti-mTOR	mTOR	Cell Signaling	2983	1:1000					
Anti-phospho-mTOR (S2448)	mTOR pS2448	Cell Signaling	5536	1:1000					
Anti-S6 Kinase	S6K	Cell Signaling	9202	1:1000					
Anti-phospho-S6 Kinase (T389)	S6K pT389	Cell Signaling	9234	1:1000					
Anti-PRAS40	PRAS40	Cell Signaling	2610	1:1000					
Anti-phospho-PRAS40 (T246)	PRAS40 pT246	Cell Signaling	2997	1:1000					
Anti-HER2	HER2	Thermo	MS-325-P0	1:200					
Anti-phospho-HER2 (Y1196)	HER2 pY1196	Cell Signaling	6942	1:1000					
Anti-EGFR	EGFR	Epitomics	1902	1:4000					
Anti-phospho-EGFR (Y1068)	EGFR pY1068	Cell Signaling	2234	1:1000					
